# – Comments on ‘mapping the evolving trend of research on class III malocclusion: a bibliometric analysis’ by Hu et al.

**DOI:** 10.1007/s00784-025-06495-y

**Published:** 2025-08-11

**Authors:** Yuh-Shan Ho, Nikolaos Christidis

**Affiliations:** 1 CT HO Trend, 3F.-7, No. 1, Fuxing N. Rd., Songshan Dist, Taipei City, 105611 Taiwan; 2 Division of Oral Rehabilitation, Department of Dental Medicine, Karolinska Institutet, Huddinge, SE-14104 Sweden

**Keywords:** Bibliometrics, Validity, Releability, Reproducibility, Accuracy, Malocclusion

## Brief summary

Hu et al. recently published a paper in Clinical Oral Investigations titled ‘Mapping the evolving trend of research on Class III malocclusion: A bibliometric analysis [1].’ The article presents a bibliometric analysis of Class III malocclusion research from 2000 to 2023, highlighting evolving trends and critical research areas within the field. Class III malocclusion, characterized primarily by an anterior crossbite due to maxillary dysplasia or mandibular prognathism, poses significant physiological, psychological, and developmental challenges, particularly among adolescents. Timely and effective treatment remains crucial, with recent developments emphasizing temporary anchorage devices and innovative methods like miniscrew-assisted rapid palatal expansion, miniscrew-anchored Forsus devices, and bone-anchored maxillary protraction facemasks.

The study aimed to map research trends and identify key focal areas through quantitative bibliometric methods using data from the Web of Science Core Collection, analyzed with tools like VOSviewer and CiteSpace. It included co-occurrence, co-citation, cluster analysis, and burst detection. Findings revealed a significant increase in publications and citations post-2015, demonstrating growing academic interest. Major contributing countries included the USA, South Korea, Japan, and China, with leading institutions such as the University of Florence and Seoul National University prominently active. Key research themes identified included orthognathic surgery, skeletal anchorage, and the increasingly popular surgery-first approach. Recent trends spotlighted a growing focus on patients’ quality of life as a critical outcome measure. In conclusion, the bibliometric analysis indicates the current landscape and shifting emphases within Class III malocclusion research. It emphasizes ongoing advancements in skeletal anchorage, surgical strategies, and the integration of quality-of-life assessments, informing future research directions and clinical practices.

## Comment on published paper

Hu et al. [1], in their study “Mapping the evolving trend of research on Class III malocclusion: A bibliometric analysis,” utilized a suboptimal search strategy, potentially leading to misleading conclusions for readers of *Clinical Oral Investigations*. This emphasizes the critical importance of applying rigorous, well-validated, and systematic search methodologies in bibliometric research. Methodological rigor and transparency are essential for ensuring the accuracy, reliability, reproducibility, and scholarly integrity of the research outcomes. Future bibliometric studies should prioritize precise, comprehensive, and clearly documented search strategies, which involve detailed reporting of search terms, databases consulted, inclusion and exclusion criteria, and selection procedures. Such meticulous methodological approaches will not only strengthen the validity of the findings but also enable other researchers to replicate and validate results effectively, thereby contributing to more robust, meaningful, and credible insights within the scholarly community. In the section of Materials and methods Hu et al. presented these search strategies:

**(1) Database**: Web of Science Core Collection; Year published (PY): 2000–2023; **(2) Document type (DT)**: article or review; **(3) Topic (TS)**: (“class III” AND malocclusion) OR (“angle class III” OR underbite OR “skeletal class III” OR “maxillary dysplasia” OR “maxillary retrusion” OR “mandibular hyperplasia” OR “mandibular protrusion” OR “mandibular prognathism”).

When it comes to the Web of Science Core Collection it includes the following:

1. Science Citation Index Expanded (SCI-EXPANDED) -- 1900-present.
2. Social Sciences Citation Index (SSCI) -- 1900-present.
3. Arts & Humanities Citation Index (A&HCI) -- 1975-present.
4. Conference Proceedings Citation Index - Science (CPCI-S) -- 1990-present.
5. Conference Proceedings Citation Index - Social Sciences & Humanities (CPCI-SSH) -- 1990-present.
6. Book Citation Index– Science (BKCI-S) -- 2005-present.
7. Book Citation Index– Social Sciences & Humanities (BKCI-SSH) -- 2005-present.
8. Emerging Sources Citation Index (ESCI) -- 2015-present.
9. Current Chemical Reactions (CCR-EXPANDED) -- 1985-present.
10. Index Chemicus (IC) -- 1993-present.

The search strategies in the original paper [1], that only included part of the Web of Science Core Collection (e.g. 8 out of 10 databases = *SCI-EXPANDED*,* SSCI*,* A&HCI*,* BKCI-S*,* BKCI-SSH*,* ESCI*,* CCR-EXPANDED*, and *IC*) resulted in 3,469 articles and reviews including 3,426 published from 2000 to 2023 were searched out from a part of the.

In 2011, Ho’s research team introduced the “front page” concept, a filtering mechanism aimed at improving search strategies within bibliometric studies. This mechanism utilizes Topic (TS) terms sourced from the Web of Science Core Collection [2], focusing on key elements such as titles, abstracts, and author keywords, to minimize the inclusion of irrelevant publications in bibliometric analyses.

After applying the “front page” filter, 3,086 of the 3,426 articles and reviews (90%) included the search keywords within their title, abstract, or author search keywords. This means 340 papers (10%) lacked these search keywords on the “front page”. Such a notable discrepancy between the total number of papers (3,426) and those meeting the “front page” criteria (3,086) raises concerns about the robustness of the filtering process in scientific research. For instance, the review titled “CBCT in orthodontics: Assessment of treatment outcomes and indications for its use” [3] and the article titled “Orthopedic traction of the maxilla with miniplates: A new perspective for treatment of midface deficiency” [4], do not include the search keywords in their title, abstract, or author keywords. This highlights a bias of relying solely on the Topic (TS) search and how significant it is to use a “front page” filter for comprehensive bibliometric analysis, as irrelevant papers may be included, which could result in misleading conclusions.

In conclusion, rigorous and transparent search methodologies are fundamental to the credibility and replicability of bibliometric research. By emphasizing precise documentation and comprehensive strategies, future studies will enhance validity, facilitate reproducibility, and generate more robust and impactful scholarly insights.

## Data Availability

No datasets were generated or analysed during the current study.
